# Gene Therapy for Bone Engineering

**DOI:** 10.3389/fbioe.2015.00009

**Published:** 2015-02-02

**Authors:** Elizabeth Rosado Balmayor, Martijn van Griensven

**Affiliations:** ^1^Experimental Trauma Surgery, Department of Trauma Surgery, Klinikum rechts der Isar, Technical University Munich, Munich, Germany; ^2^Institute for Advanced Science, Technical University Munich, Garching, Germany

**Keywords:** gene therapy, bone regeneration, bone morphogenetic proteins, hydrogel, sonoporation, adenovirus

## Abstract

Bone has an intrinsic healing capacity that may be exceeded when the fracture gap is too big or unstable. In that moment, osteogenic measures need to be taken by physicians. It is important to combine cells, scaffolds and growth factors, and the correct mechanical conditions. Growth factors are clinically administered as recombinant proteins. They are, however, expensive and needed in high supraphysiological doses. Moreover, their half-life is short when administered to the fracture. Therefore, gene therapy may be an alternative. Cells can constantly produce the protein of interest in the correct folding, with the physiological glycosylation and in the needed amounts. Genes can be delivered *in vivo* or *ex vivo* by viral or non-viral methods. Adenovirus is mostly used. For the non-viral methods, hydrogels and recently sonoporation seem to be promising means. This review will give an overview of recent advancements in gene therapy approaches for bone regeneration strategies.

## Introduction

Bone tissue can heal relatively well in a natural way. A defect in cortical bone will spontaneously heal if the gap is smaller than 2 mm. A prerequisite for bone healing is absolute fracture stability (Gaston and Simpson, 2007). Unfortunately, trauma, bone tumor resections, or arthritis may lead to larger bone defects that may have a compromised healing. Delayed healing or non-union occurs in 5–10% of all fractures and 20% of high impact fractures (Brydone et al., 2010). This impaired healing is caused by the body’s inability to regenerate the bone and additional surgical interventions, besides stabilization, may be needed to replace the lost bone. Autografts, allografts, and bone grafts substitutes are mainly used for this purpose in an attempt to fill these non-healing bone defects. Osteogenic growth factors may also be added to the bone graft substitutes in order to kick-start or accelerate bone healing. More recently, autologous stem cells derived from bone marrow have been administered to enhance the healing of the non-union bone defects.

Thus, the important prerequisites for bone healing are: (i) cells with osteogenic potential, (ii) osteoconductive matrix, (iii) osteoinductive stimulus, and (iv) a mechanical stable environment (Giannoudis et al., 2007). The authors named this the “diamond concept” and all components must be active for a successful bone-union to occur.

**Figure 1:**
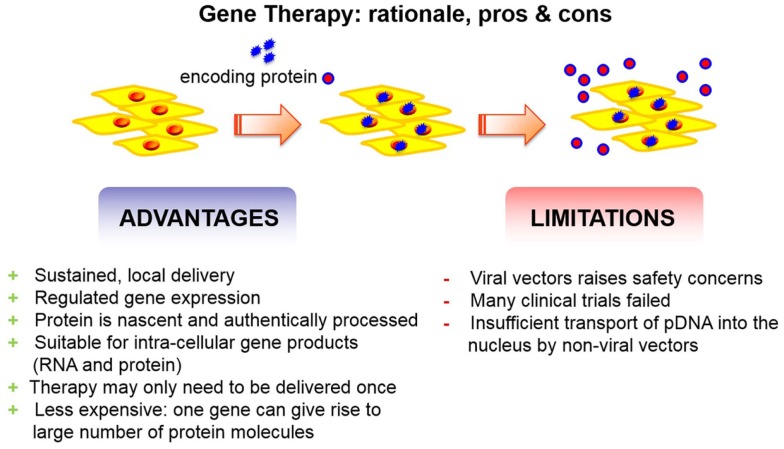
**Gene therapy has advantages and disadvantages**. The advantages outreach the limitations.

Recombinant growth factors, however, are expensive and cumbersome to produce. This is because, eukaryotic cells are needed in order to have a correct folding as well as glycosylation of the protein. Furthermore, once transferred to the body, the growth factors have a short half-life and need to be administered in high supraphysiological concentrations. Therefore, gene therapy may be an alternative (Figure 1). Indeed, there are several advantages of gene delivery over protein delivery, which are well supported by a fair number of scientific studies. The most relevant advantages of gene therapy include the flexibility to express the protein locally and focally, or in a disseminated fashion, as needed. Of note, gene therapy brings the possibility for intra-cellular production of proteins. Thus, this facilitates therapeutic pathways to take place. Unlike its recombinant equivalent, the protein delivered via gene transfer will be nascent and uncontaminated by a variable percentage of incorrectly folded and possibly antigenic molecules (Evans, 2012). Moreover, additional advantages of gene delivery include the ability to express proteins for extended periods of time and the level of transgene expression can be regulated.

A second aspect of the need of high protein doses is possible side effects that may hamper safety of the therapy. Adverse events for the use of BMP-2 mainly in spinal fusion are ectopic bone formation, swelling, seroma, retrograde ejaculation, dysphagia, and tumor formation (Woo, 2012; Fu et al., 2013). Swelling due to use of BMP-2 in anterior cervical spine fusion was observed in 28% of the patients (Smucker et al., 2006). In anterior interlumbar interbody fusion, several adverse events may occur. Retrograde ejaculation occurs in 6.3–7.4% of the patients, which may lead to a two times increased incidence of urinary retention (Carragee et al., 2011; Comer et al., 2012). A controversy exists concerning cancer risk upon BMP-2 administration. Several studies conclude that no increased cancer risk occurs when BMP-2 is used for spinal arthrodesis (Cooper and Kou, 2013; Kelly et al., 2014). However, Carragee et al. (2013) reported that high doses of BMP-2 in spinal arthrodesis result in a five times increased cancer risk 2 years after surgery.

Thus, gene therapy often reduces the amounts of therapeutic molecules. It may only need to be delivered once and in a relatively small amount (Evans, 2012). Thereby, the adverse events described above may not occur. The cancer risk e.g., was only increased when high doses of BMP-2 were used and by using gene therapy, only low amounts of protein are produced not leading to this increased cancer risk. In addition to its therapeutic potential, gene delivery is a valuable experimental tool for laboratory research into the biology of bone. Translating these facts to the osteology area, gene transfer to bone has demonstrated its huge therapeutic capabilities. As a matter of fact, gene transfer using viral vectors has already shown that bone healing and treatment of other bone disorders such as bone tumors or osteogenesis imperfecta can be possible. Although limitations are associated with viral vectors, studies clearly reveal the strong advantages of using gene therapy over treatments with the recombinant protein. Indeed, integration occurs even with integration non-competent viruses such as adeno-associated viruses (Kaeppel et al., 2013). However, this did not lead to tumorigenicity. Another important issue is the fact that preexisting antibodies or memory T-cells may diminish the efficacy of AAV gene therapy (Mingozzi and High, 2013). To screen patients beforehand may improve the effectiveness. However, many patients may than be excluded as AAV are commonly encountered in normal life and thereby an immune-memory has developed. Nevertheless, there is a believable proof of principle in both *in vitro* and animal model experiments that gene transfer can be successfully used to regenerate bone (Evans, 2012).

### History of gene therapy

The first idea related with a gene therapy approach evolved as early as 1966 and was mentioned by Edward Tatum when he speculated that viruses could be used effectively to introduce new genes into defective cells of particular organs (Tatum, 1966). Tatum also suggested the first definition of a field that was called “human genetic engineering” at that time. He defined human genetic engineering as the alteration of existing genes in an individual and stated that the first successful genetic engineering would be performed with the patient’s own cells (Tatum, 1966). One year later, Lederberg mentioned the term “virogenic therapy” in a publication in the Washington Post in which he defended the idea that viruses could be used to transfer DNA molecules that could encode for a therapeutic entity into cells of patients suffering from hereditary defects (Lederberg, 1968). In 1969, the first isolation of a gene succeeded by Beckwith (1969) promising a brilliant future to the so-called human genetic engineering. However, growing debates on social and ethical implications accompanied the field throughout the 1960s and 1970s. The gene therapy concept was criticized as being remote and improbable, even unnecessary. Several prominent scientists rejected all the rationale behind gene therapy and the use of DNA with therapeutically aims (Burnet, 1971). Together with this hostile background, Stanfield Rogers failed, when he performed the first attempt at human gene therapy, in the late 1960s. He injected the Shope papilloma virus into patients with arginase deficiency. His assumption that the virus contained an arginase gene and that would induce arginase expression or leads to the preferential growth of cells with higher arginase activity, could not be proven. The treated patients did not show any effect on their arginase levels after injection of the virus. In 1980, a second attempt is registered when Cline and colleagues tried to transfect the β-globin gene into human bone marrow cells. The cells were subsequently transplanted into patients suffering from thalassemia. Their trial was criticized for both scientific and procedural reasons (Wolff and Lederberg, 1994). Both trials lacked a sound practice and well-proven cell culture and animal experiments.

It was not until the development of recombinant DNA technology together with early transfection and cell culture techniques that major progress was made in gene transfer. Subsequently, several disease-related genes (e.g., herpes TK gene, APRT, and human HPRT) were successfully transferred into mammalian cells proving the feasibility of the technique. Therefore, the first approved gene therapy case took place at the NIH for treating a genetic defect that caused a severe immune system deficiency (ADA-SCID) in 1990. The results were successful, however temporary. Up to the present, a fair number of clinical trials for chronic and acute lymphocytic leukemia, multiple myeloma, thalassemia, coronary artery disease, HIV, and retinal diseases among others have been or are being conducted using a gene therapy approach.

## Delivery Platforms for Gene Therapy for Bone Engineering

Despite the above-mentioned advancements, transfections of bone-related cells, bone-derived stem cells, or bone tissue aiming to bone regeneration have hardly been performed. Different strategies exist to perform gene therapy for bone engineering:

1. *In vivo*
  - Viral
  - Non-viral
2. *Ex vivo*
  - Viral
  - Non-viral

In the *in vivo* approach, the vector (viral or non-viral) is administered to the fracture gap and resident cells are expected to be transfected (van Griensven et al., 2002). They will locally produce the osteogenic protein. The administration can be via direct injection or associated with a biomaterial. The latter combination of vector and biomaterial is called gene activated matrix (GAM). Regarding the *ex vivo* approach, one does not rely on the cells to be transfected *in situ*. Autologous cells will be harvested [e.g., mesenchymal stem cells from bone marrow (BMSC) or adipose tissue (AdMSC)] and transduced outside the body. The transduced cells are subsequently implanted in the fracture gap. Again, direct injection or using a biomaterial as carrier is the main method of application.

### Viral gene transduction

Viruses are widely used as their mode of action is to transfect mammalian cells with their genetic material. Most used virus types for gene therapy are adenoviruses, adeno-associated viruses, lentiviruses, and retroviruses. Recombinant viral vectors are widely used. It has the ability to infect different cell types with high efficiency. No differences in efficiency are reported for using dividing or non-dividing cells. The gene of interest is not incorporated in the human genome and will be non-detectable after several cell cycles. Reports in bone regeneration mainly employ adeno- or retrovirus vectors carrying plasmids that encode for bone morphogenetic proteins (BMPs) (Park et al., 2003; Tsuda et al., 2003). In addition, GAM have been also used *in vivo* for bone healing. Those are mainly based on the loading of BMPs plasmid/viral vectors complexes onto biomaterials (e.g., collagen, chitosan, polyesters, and calcium phosphates) to be implanted at the defect site (Chang et al., 2010; Zhang et al., 2011).

Rat femoral defects have been treated with adenoviral constructs encoding BMP-2, Runx2, or VEGF. BMP-2 healed the femoral defects dose-dependently (Betz et al., 2007b) upon direct percutaneous injection (Betz et al., 2006). When the authors did not immediately apply the vector, but performed delayed injection, the results were even more pronounced (Betz et al., 2007a). When performing a GAM approach using transduced muscle or adipose grafts, no difference could be obtained with autograft (Evans et al., 2009; Betz et al., 2013). Similar effects were obtained with a hydrogel formulation (Sonnet et al., 2013). When using MSC transduced with adenoviral BMP-2, efficient healing could also be detected (Lieberman et al., 1999; Peterson et al., 2005). Using the more downstream runx2 signal transduction molecule within an adenoviral vector, induced higher bone mineral density upon direct injection in the bone marrow of a rat femur (Bhat et al., 2008). However, not only osteogenic genes result in improved fracture healing, also inducing angiogenesis by a VEGF-adenoviral vector was able to promote bone formation (Tarkka et al., 2003).

Besides rat studies, also larger animals are used such as rabbits, sheep, and pig. The latter is mainly studied for calvarial defects. A rabbit femur segmental defect could be healed by injection of a BMP-2 encoding adenoviral vector (Baltzer et al., 2000). Also in sheep, this treatment was successful (Egermann et al., 2006b). Even when the sheep were osteoporotic, the BMP-2 could induce fracture healing (Egermann et al., 2006a). Goat have similar physiologic properties as sheep. Tibial defects in goats were treated with a scaffold composed of biphasic calcined bone and autologous BMSC transduced with human BMP-2. Five goats showed complete healing and three partial healing after 26 weeks (Dai et al., 2005). However, a temporary cellular and persistent humoral immune responses against adenovirus could be detected (Xu et al., 2005).

### Non-viral gene therapy for bone engineering

Despite all the above-mentioned restraints, viruses currently remain the carriers of choice in most of the gene therapy studies and clinical trials. However, safety concerns are continuously raised associated with their use. This is based on the fact that they naturally transfer their genetic material very efficiently into the cells. For viral gene therapy, the viral genome is modified by removing the sequences that contribute to their pathogenicity (Evans, 2012). However, the safety concerns are constantly growing together with the fact that viral vectors can be expensive and their production is complicated (Schleef et al., 2010; Elsabahy et al., 2011).

Therefore, high interest has been placed in the use of non-viral vectors during the last two decades. Cationic polymers, lipids, peptides and even calcium phosphate, and other inorganic nano-materials have been explored for their capabilities as carriers of genetic information into a target cell for *in vivo* gene therapy (Loh and Lee, 2012). Among them, cationic liposomes and cationic polymers are by far the most widely utilized carriers for gene and nucleic acid delivery today (Tros de Ilarduya et al., 2010; Won et al., 2011). Because of their opposite surface charge, they are commonly utilized for gene transfer by forming a complex (lipoplexes or polyplexes) with negatively charged DNA molecules. A common disadvantage of those systems is their still relatively low transfection efficiency when compared to viral vectors, especially when “difficult-to-transfect cells” such as MSCs represent the target cell. Although it is worth mentioning that progress in lipid development has achieved quite satisfactory levels of transfections in recently published studies (Jain et al., 2013; Locatelli et al., 2013; Sarker et al., 2013). Unfortunately, they often have toxic effects on the cells. Both cationic lipids and polymers are not biodegradable and therefore, the risk of their accumulation in the body is high. Based on all the aforementioned facts, it can be concluded that the development of highly efficient and less toxic gene carriers is the most challenging work in the field of non-viral gene therapy (Medina-Kauwe et al., 2005; Shan et al., 2012).

The work of Tomas’ group is encouraging, demonstrating a successful transfection of adipose tissue-derived MSCs with a G4 PAMAM/BMP-2 plasmid dendriplex inducing this cells to differentiate into the osteogenic phenotype, even when only low transfection efficiencies were achieved (Santos et al., 2009). Also delivering BMP-2 cDNA in an alginate hydrogel is promising. Biologically active BMP-2 is released from the BMSC present in the gel over a period of 5 weeks. This leads *in vivo* to ectopic osteogenesis (Wegman et al., 2011). Other hydrogels such as fibrin or hyaluronic acid may also be used as carriers for nucleic acid vectors (Schillinger et al., 2008; des Rieux et al., 2009; Lei et al., 2010, 2011). They can be used for delivering osteogenic genes and induce bone formation and accelerate fracture healing (Yang et al., 2012; Kaipel et al., 2014).

Another novel method for transducing cells is the so-called sonoporation (Mehier-Humbert et al., 2005; Li et al., 2009). Ultrasound is used in combination with microbubbles to transfect cells. Therefore, a novel osteoinductive non-viral *in vivo* gene therapy approach using sonoporation was investigated in ectopic and orthotopic models (Sheyn et al., 2008; Feichtinger et al., 2014b). BMP-2 and BMP-7 co-expression plasmids were repeatedly applied for 5 days with or without sonoporation. Transduction efficiency was observed using a luciferase plasmid and bioluminescence imaging in an ectopic model. Luminescence demonstrated increased transduction efficiency in sonoporated animals in comparison with passive gene delivery (Feichtinger et al., 2014b). Using osteogenic plasmids like BMP-2/BMP-7 or BMP-9, enhanced ectopic bone formation was detected for sonoporation compared to passive gene delivery (Sheyn et al., 2008; Osawa et al., 2009; Feichtinger et al., 2014b). Also orthotopic application in a rat femur non-union model demonstrated similar results using sonoporation. Sonoporated animals showed an increased union rate (Feichtinger et al., 2014b).

## Novel Approaches Other than Growth Factors

Besides the use of genes for BMP or other growth factors, much attention has been given recently to miRNA use with therapeutic aims. miRNAs are small (approximately 20 nt), non-coding RNAs. Over 4,000 miRNAs have been identified so far in the human genome. They regulate many biological processes in the human body. They are known to adjust and switch regulatory circuits governing tissue repair. Key elements of tissue repair such as stem cell biology, inflammation, and angiogenesis are under control of a network of miRNAs (Sen, 2011). They were first discovered associated with cancer treatments and cardiovascular diseases. Recently, several miRNAs have been identified so far to be associated with bone pathologies (Seeliger et al., 2014). For example, miRNA-218 has been reported to be a pro-osteoblastic factors by acting on Wnt inhibitors. A DNA aptamer that binds sclerostin has a similar activity (Shum et al., 2011). miRNA-148a is a pro-osteoclastic factor by blocking MAFB signaling (van Wijnen et al., 2013). In addition to miRNA-218, 10 other miRNAs have been identified to control osteoblast differentiation and are expressed in osteoblastic cells (i.e., miRNA-23a, miRNA-30c, miRNA-34c, miRNA-133a, miRNA-135a, miRNA-137, miRNA-204, miRNA-205, miRNA-217, and miRNA-338) (van Wijnen et al., 2013). From a therapeutic perspective, *in vivo* approaches that promote the activity of pro-osteoblastic miRNA or inhibit pro-osteoclastic miRNAs are highly attractive for stimulating bone formation. However, efficient tools for delivering those miRNA mimics (e.g., to stimulate pro-osteoblastic miRNA) or inhibitors (e.g., to block pro-osteoclastic miRNAs) to specific target tissue are limited. Two possibilities were shown by Li et al. They could show that transfected MSCs with miRNA26a in hydroxalapatite–tricalcium phosphate scaffolds induced bone formation subcutaneously. Furthermore, a hydrogel delivery system consisting of HyStem-HP could enhance bone regeneration in a rat calvarial model (Li et al., 2013). Using miRNA transfected MSCs is common. Transfection of MSCs with miRNAs can be improved using magnetic nanoparticles (Schade et al., 2013, 2014). miRNA-31 transfected MSCs in a polyglycol sebacate scaffold accelerated and improved the healing of a rat critical-size calvarial defect (Deng et al., 2014). miRNA-138 transfected MSCs on a cell sheet and implanted subcutaneously in immunocompromised mice showed bone formation (Yan et al., 2014).

Besides the modulating miRNA, mRNA can be blocked using silencing RNA (siRNA). Depending on the target, osteogenesis can be promoted. Using siRNA against glucocorticoid receptors encapsulated in poly(lactid-co-glycolic acid) resulted in an upregulation of alkaline phosphatase and RunX2 in MSCs (Hong et al., 2012). Similar results on these two genes were obtained using siRNA against Noggin delivered in PEG hydrogels (Nguyen et al., 2014). The RunX2 pathway is very important for osteogenesis and the siRNA against guanine nucleotide-binding protein α-stimulating activity polypeptide 1 is modulating this. It was shown that in MSCs treatment with this siRNA lead to upregulation of collagen type I, osteopontin, and alkaline phosphatase (Rios et al., 2012). This siRNA and siRNA against prolyl hydroxylase domain-containing protein 2 increased bone volume when implanted in a fibroin–chitosan cage above ovine periosteum *in vivo* (Rios et al., 2012). This biomaterial seems favorable for the delivery of siRNA. Chitosan sponges were loaded with siRNA against casein kinase 2 interaction protein 1 and soluble vascular endothelial growth factor receptor 1. This induced expression of osteocalcin, alkaline phosphatase, and vascular endothelial growth factor in MSCs. Moreover, mineralization as evidenced by alizarin red staining was increased. Furthermore, administration in a critical-size calvarial model resulted in accelerated, complete bone regeneration (Jia et al., 2014). Finally, siRNA against LNK protein accelerated femur fracture healing in a mouse model. This was accompanied by increased osteoblast activity (Kawakami et al., 2013).

### Optimizing delivery, expression, and function

All of the above-mentioned novel approaches need optimization. The genes need to be locally expressed in specific target cells (spatial control). This may be achieved by using aptamers that specifically bind osteogenic progenitor cells (Ardjomandi et al., 2013). Another possibility is using a tissue specific promotor for conditional expression of the gene of interest (Lian et al., 2000). Furthermore, the level of gene expression needs to be controlled. This may be achieved by using molecular sensors and negative feedback loops (Kaempfer, 2003). Moreover, the period of expression needs to be controlled. This may be achieved by using TET-on/TET-off systems and administration of doxycycline (Feichtinger et al., 2014a). Finally, angiogenesis is important for bone regeneration. Combining osteogenic and angiogenic factors may enhance bone formation as shown already in the siRNA approach against guanine nucleotide-binding protein α-stimulating activity polypeptide 1 and prolyl hydroxylase domain-containing protein 2 (Rios et al., 2012). But also BMP and vascular endothelial growth factor gene co-delivery may enhance osteogenesis (Huang et al., 2005; Samee et al., 2008; Wu et al., 2012).

## Conclusion

Today, the scientific community worldwide has identified main problems and limitations behind the current gene therapy approach. However, it remains undoubted that a safe and efficient gene therapy approach could have a huge impact on non-curable diseases today. Therefore, a fair number of scientific efforts are aimed to find solutions and optimize this approach. Currently, we are aware of the need for a therapeutically functional DNA after it has been transported to the nuclei. The risks of stimulating the immune system in a way that the effectiveness of the therapy will be diminished are always present. The chances for tumor formation as a consequence of a wrongly integrated DNA have also occurred in earlier clinical trials. Viruses represent one of the most efficient means to transfer the genetic information to the cells. However, they also carry a variety of strong limitations. Some examples are toxicity, immune responses, and the potential risk for recovering their ability of causing diseases.

Gene therapy for bone regeneration is important and should be further investigated. Combinations of different genes in association with biomaterials are in our opinion the most promising to bring the field forward. At the moment, one clinical trial has just started November 11th 2014 with a GAM based on collagen-hydroxyapatite including the gene for vascular endothelial growth factor-A165 to treat alveolar bone loss (clinicaltrials.gov: NCT02293031). No other clinical trials are ongoing at the moment. We think that in the near future gene therapy for bone regeneration will not be implemented in the clinical arena. However, as science and technology progress, clinical translation is not out of reach.

## Conflict of Interest Statement

The authors declare that the research was conducted in the absence of any commercial or financial relationships that could be construed as a potential conflict of interest.
